# Environmental factors between normal and superagers in an Argentine cohort

**DOI:** 10.1590/1980-57642020dn14-040003

**Published:** 2020-12

**Authors:** Ismael Luis Calandri, Lucia Crivelli, Maria Eugenia Martin, Noelia Egido, Nahuel Magrath Guimet, Ricardo Francisco Allegri

**Affiliations:** 1 Departamento de Neurología, FLENI Ringgold Standard Institution – Buenos Aires, Argentina.; 2 Universidad de la Costa – Barranquilla, Atlántico, Colombia.

**Keywords:** cognitive aging, healthy aging, aging, neuropsychology, envelhecimento cognitivo, envelhecimento saudável, envelhecimento, neuropsicologia

## Abstract

Normal aging usually brings age-related cognitive decline. However, there is a group of aged individuals who have exceptional memory performance: the superagers.

**Objective:**

Our aim was to identify the environmental factors that could influence exceptional memory performance in a cohort of Argentine individuals.

**Methods:**

Forty healthy volunteers >80 years of age were classified into two groups, superagers (SA, n=20) and normal agers (NA, n=20), according to the Northwestern SuperAging Program criteria. Participants were neuropsychologically tested and evaluated on environmental aspects: working status, education, bilingualism, cognitive reserve, physical activity, social networking, clinical comorbidities, and longevity of parents and siblings.

**Results:**

Both groups were highly educated (NA=16.3±3 years; SA 15.85±2.6; p=0.6), 11.8% of the sample was still working without differences between groups. There were no differences in cognitive reserve inventory (p=0.7), physical activity engagement (p=0.423), or social network index (p=0.73). As for longevity, 44% of the siblings lived longer than 80 years of age (p=0.432) and maternal longevity was linked to SA (NA=46.7%; SA=80%; p=0.045).

**Conclusions:**

This study is a pilot approximation to the superaging population in Argentina. Our results suggest that environmental factors related to successful aging do not differentiate superaging. SA may depend on variables yet to be identified, probably of a genetic/metabolic order.

## RESUMO.

O envelhecimento normal geralmente traz declínio cognitivo relacionado à idade. No entanto, existe um grupo de idosos com desempenho excepcional de memória: os superidosos.

**Objetivo:**

Nosso objetivo foi identificar os fatores ambientais que podem influenciar o desempenho excepcional da memória em uma coorte de indivíduos argentinos.

**Métodos:**

Quarenta voluntários saudáveis com idade >80 anos foram classificados em dois grupos: superidosos (SI, n=20) e idosos normais (IN, n=20), de acordo com os critérios do Programa de Superidosos da Universidade Northwestern. Os participantes foram testados neuropsicologicamente e avaliados nos aspectos ambientais: situação de trabalho, escolaridade, bilinguismo, reserva cognitiva, atividade física e rede social, comorbidades clínicas e longevidade de pais e irmãos.

**Resultados:**

Ambos os grupos tinham alto nível de escolaridade (IN=16,3±3 anos; SI=15,85±2,6; p=0,6), 11,8% da amostra ainda trabalhava sem diferença entre os grupos. Não houve diferenças no inventário de reserva cognitiva (p=0,7), prática de atividade física (p=0,423) ou índice de rede social (p=0,73). Quanto à longevidade, 44% dos irmãos viviam mais de 80 anos (p=0,432) e a longevidade materna estava associada a SI (IN=46,7%; SI=80%; p=0,045).

**Conclusões:**

Este estudo é uma aproximação piloto ao super envelhecimento da população na Argentina. Nossos resultados sugerem que os fatores ambientais relacionados ao envelhecimento bem-sucedido não diferenciam os superidosos. Ser superidoso pode depender de variáveis ainda não identificadas, provavelmente de ordem genética/metabólica.

## INTRODUCTION

Life expectancy increase calls for an in-depth understanding of the factors that can optimize processes, whether normal or pathological. Aging, as other stages of life, brings changes in cognition. Thus, age-related cognitive changes are well-documented in the literature.^1^ Thus, while some cognitive skills tend to improve with age — in particular, cognitive abilities associated to crystallized intelligence, and knowledge cumulation based on life experience, such as vocabulary and general knowledge, other skills associated to fluid intelligence — such as problem-solving and manipulation of information — tend to decrease with age, as reflected by a decrease in processing speed and memory with age.^2^ ^,^ ^3^

Notwithstanding this evidence, the literature reports on a group of aging subjects, whose performance on cognitive tasks is superior to what is expected for their age. There have been multiple attempts to define and characterize this population. Some studies refer to this group as “High-performing older adults” (HPOA)^4^ ^,^ ^5^ and others as Superagers (SA).^6^ The operative definition of superging was used in this work, in order to refer to subjects aged over 80 years old with cognitive performance at least 20 years younger.^7^

To date, there is no consistent evidence about the factors that make these subjects’ cognitive performance exceptional. One of the working hypotheses concerns the potential role of environmental factors such as education, bilingualism, cognitive reserve, and social networks in cognitive preservation. Along this line, a study by Maher et al.^8^ found that SA had the same level of psychological well-being as normal controls, but endorsed greater levels of positive social relationships. Another complementary hypothesis was assessed in an interesting review that revealed that HPOA have structural and molecular preservation of the brain ‘cortex, especially the anterior cingulate region, and hippocampal volumes greater than normal aging subjects.^9^

A recent study by Borelli et al.^10^ made the first approach to characterize HPOA in Latin America revising and adapting the criteria, including a revision of the implication of low education that is frequent in Latin America. The study states that low education may mask or cover certain exceptional cognitive performing subjects. Thus, cognitive tools and normative sources for this tool should be selected carefully. Also, regarding the role of education in healthy subjects over 80 in Latin America, a study by Balduino et al.^11^ found that the differences in cognitive performance are strongly related to education and that the study of SA should include an analysis of the educational level.

The aim of the present study was to examine the potential role of environmental factors in the SA performance of an unstudied cohort of Latin American individuals. Our hypothesis is that factors frequently associated with cognitive reserves and healthy aging could be significantly stronger in the SA group than in normal aging subjects.

## METHODS

### Participants

Forty participants were recruited within volunteers over 80 years of age at the memory and aging clinic in Fleni, Buenos Aires. The local ethics committee approved the protocol and all subjects signed an informed consent form prior to assessment. The criteria for participants were absence of memory complaints, full independence in activities of daily living, and normal cognition according to patients and their family. Normal cognition was assessed using an comprehensive neuropsychological evaluation (including UDS 2 battery,^12^ ^,^ ^13^ Rey Auditory Verbal Learning Test (RAVLT),^14^ phonological fluency and Rey Osterreith Figure Test. Subjects were classified into Normal Aging (NA) (n=20) and SA (n=20) according to the Northwestern University SuperAging Program criteria.

#### Criteria for Superaging and Normal aging groups

According to the Northwestern University SuperAging Program,^7^ SA must present a normal performance (*i.e.*, above −1 SD in relation to the mean) by reference to normative values of individuals two decades younger (age range from 50 to 60 years old) on delayed recall score of the RAVLT and a normal score for their age range on the 30-item Boston Naming Test (BNT);^15^ the Trail Making Test Part B,^16^ and the Semantic Fluency Test.^17^

To be classified as NA group, subjects had to be considered normal for their normative age value (*i.e.*, above −1 SD in relation to the mean) in the same tests and not fulfill the SA criteria.

We used the *rioplatense* Spanish version of the RALVT and normative values of the Memory and Ageing Center at Fleni database with 1,000 normal individuals divided by age range and educational level.

#### Individual factors

History of age-related chronic diseases (as hypertension, diabetes, and dyslipidemia), and the presence of clinical illness and major surgeries were considered in both groups.

Furthermore, the Cognitive Reserve Inventory,^18^ was administered to all participants, given that cognitive reserve is considered a protective factor against age-related cognitive decline.

#### Parental-family factors identification

In order to test possible genetic factors related to SA emergence, we evaluated the longevity of parents (age of death) and siblings (percentage of siblings older than 80 years of age), and subject's report of first-degree family history of significant memory impairment.

#### Lifestyle factors

Lifestyle factors having shown to impact cognitive aging were measured with the Social Network Index^19^ (Network size and Diversity sub-scores), including information such as the age of retirement, currently job, and marital status.

Social involvement was measured through the Social Network Index (Network size and Diversity sub-scores). Physical activity was measured according to the WHO criteria for sedentarism in daily life.^20^

#### Data analysis

Statistical analysis was performed by the *Statistical Package for the Social Sciences* (SPSS), version 24.0.

Continuous variables were tested for normality of distribution by Kolmogorov-Smirnov's test. Differences between groups were assessed by means of the t-test or the Mann-Whitney test according to normality diagnosis. For categorical variables, we used the chi-square test over a two-tailed hypothesis. A significant statistical level was established at p<0.05. In order to control the family-wise error rate, the Benjamini and Hochberg^1^ method was applied, the adjusted p-values are reported.

## RESULTS

### Individual factors

No gender or age differences were found (mean age=82.8±2.49, 36% females).

Both groups were equally educated (NA=16.3±3 years; SA 15.85±2.6 p=0.6) with no differences between groups. High frequency of bilingualism was found (NA=81.3%, SA=80% p=0.631), and 30% of NA and 33% of SA dominated more than 3 languages, there were no differences in the cognitive reserve inventory (p=0.7). There was no significant difference in the prevalence of cardiovascular risk factors (p=0.481) 47.2% of the sample lacked clinical comorbidities and 36.1% had only one. Table 1 summarizes these findings

**Table 1:** Individual factors.

	Normal aging		Superaging		sig
Age (mean [CI])	83.29	[81.96–84.63]	82.45	[81.35–83.55]	0.65
Sex (male %)	76.50		55.00		0.155
Dexterity (%)	100		100		//
Education years (mean [CI])	16.29	[14.61–17.98]	15.85	[14.62–17.08]	0.306
Speak more than one language (%)	81.30		80.00		0.631
Proficient bilingualism (%)	43.80		55.00		0.369
Cognitive reserve inventory	12.63	[10.85–14.4]	13.3	[12.07–14.53]	0.501
Hypertension (%)	25		35		0.391
Dyslipidemia (%)	31.30		15		0.223
Smoker (%)	12.50		5		0.415
Diabetes (%)	12.50		5		0.415
***Absence of CV risk factors***	43.80		50		0.481
***More than 1 CV risk factors***	25.00		10		0.398
Hypotiroidism (%)	12.50		5		0.415
Major surgeries (%)	54.50		33.30		0.23

CI: confidence interval; CV: cardiovascular; sig: statistical significance.

### Family factors

Table 2 summarizes first-degree family factors. Longevity was a frequent finding, 44% of the siblings lived longer than 80 years. There were no significant differences in group comparison (p=0.432). Significant differences were found in parental longevity, mother longevity was present in 46.7% of NA and in 80% of SA (p=0.045 and Cohen's d= −0.6411539 95CI%[-1.35, 0.07].

**Table 2:** Family and lifestyle factors.

Family factors	Normal aging		Superaging			Sig.
Mother's age of death. years old (mean [CI])	76.27	[69.43–83.11]	84.45		[78.34–90.56]	0.045
Father's age of death. years old (mean [CI])	67.14	[58.94–75.44]	73.79		[66–81.5]	0.231
Siblings over 80 years of age (percent)	0.526	[0.187–0.864]	0.389		[0.197–0.58]	0.432
Lifestyle factors						sig
Currently working	11.80%		15.00%			0.58
Retirement age (mean [CI])	71.6	[63.81–79.39]	70.95	[66.18–75.72]		0.875
Current physical activity	56.30%		65.00%			0.423
Living as a couple	58.80%		47.40%			0.363
Network diversity/social integration	4.82	[3.86–5.79]	5	[4.23–5.77]		0.763
Social network size	22.53	[16.19–28.87]	24.5	[18.21–30.79]		0.647

CI: confidence interval; sig: statistical significance.

### Lifestyle factors

There were no differences in the social network index (network diversity p=0.36, network size p=0.73). 75% of NA and 95% of SA currently engaged in physical activity (p=0.085), 11.8% still worked and 60% continued working over the standard retirement age without differences between groups.

## DISCUSSION

The aim of this study was to assess the potential role of the presence of a series of well-known protective factors against cognitive decline and dementia, as a thinkable explanation of SA's performance, in a group of healthy Latin American elderly. Results showed that both groups had a high prevalence of these factors, without significant differences between groups. The only variable that was significantly different between groups was higher longevity on the mother of SA.

To contextualize this finding, it is important to highlight some factors. First of all, the emergence of SA is always an exceptional circumstance and is even rarer in Latin America, where life expectancy is shorter than in developed countries. Large recruitment in this scenario becomes very difficult. Secondly, we proposed to measure environmental variables which are difficult to operationalize.

In line with previous works^20^ the results of the present study indicate the aforementioned factors are key to overcome the 80-year-old barrier without cognitive decline, but they cannot explain the SA status. If the environment is not strong enough to justify superaging, there must be a veiled factor conditioning its emergence. Our work could suggest some clues in that direction. Maternal longevity differences between groups suggest that there is a genetic substrate that could be necessary for superaging. These results should be interpreted as a trend due to the small sample size explored. However, the fact that longevity results apply only to maternal affiliation may be explained by the fact that the parents of these subjects lived during a particularly aggressive historical moment with the male population (*i.e.*, two world wars and cardiovascular diseases without treatment).

This study is relevant because it highlights the fact that surperaging is not an isolated phenomenon described in Northern countries, but is also present in Latin American societies. Furthermore, our results suggest that a healthy lifestyle is a necessary condition to exceed the age of 80 with normal aging, but does not explain superaging emergence. Superaging is a *rara avis* and our work remarks on the importance of inquiring on the role of genetic factors.

### Limitations and future directions

As previously stated, the recruitment of exceptionally successful older adults becomes more difficult in the context of a low life expectancy. A larger sample size would undoubtedly contribute to more solid conclusions.

High educational level is a bias in our sample. Both groups had high educational level, and no significant differences were found in this variable, leading to the assumption that education is not a direct cause of exceptional memory performance. However, this hypothesis should be further tested with a more diverse sample, including different educational levels.

Future studies should study this population longitudinally in order to address cognitive evolution and to answer a key question: is this population cognitively stable or are they declining at the same range as normal agers, though having started from a higher performance level?^21^
